# Synthetic plant defense elicitors

**DOI:** 10.3389/fpls.2014.00804

**Published:** 2015-01-26

**Authors:** Yasemin Bektas, Thomas Eulgem

**Affiliations:** ^1^Center for Plant Cell Biology, Institute for Integrative Genome Biology – Department of Botany and Plant Sciences, University of CaliforniaRiverside, CA, USA; ^2^Department of Biology, Faculty of Arts and Science, Gaziosmanpasa UniversityTokat, Turkey

**Keywords:** plant activators, systemic acquired resistance, plant innate immunity, pesticide, crop protection, salicylic acid, chemical genetics, plant defense

## Abstract

To defend themselves against invading pathogens plants utilize a complex regulatory network that coordinates extensive transcriptional and metabolic reprogramming. Although many of the key players of this immunity-associated network are known, the details of its topology and dynamics are still poorly understood. As an alternative to forward and reverse genetic studies, chemical genetics-related approaches based on bioactive small molecules have gained substantial popularity in the analysis of biological pathways and networks. Use of such molecular probes can allow researchers to access biological space that was previously inaccessible to genetic analyses due to gene redundancy or lethality of mutations. Synthetic elicitors are small drug-like molecules that induce plant defense responses, but are distinct from known natural elicitors of plant immunity. While the discovery of some synthetic elicitors had already been reported in the 1970s, recent breakthroughs in combinatorial chemical synthesis now allow for inexpensive high-throughput screens for bioactive plant defense-inducing compounds. Along with powerful reverse genetics tools and resources available for model plants and crop systems, comprehensive collections of new synthetic elicitors will likely allow plant scientists to study the intricacies of plant defense signaling pathways and networks in an unparalleled fashion. As synthetic elicitors can protect crops from diseases, without the need to be directly toxic for pathogenic organisms, they may also serve as promising alternatives to conventional biocidal pesticides, which often are harmful for the environment, farmers and consumers. Here we are discussing various types of synthetic elicitors that have been used for studies on the plant immune system, their modes-of-action as well as their application in crop protection.

## INTRODUCTION

### THE PLANT IMMUNE SYSTEM

Plants serve as a source of nutrients for a wide variety of heterotrophic microorganisms that can cause diseases in their hosts. Physical barriers, such as a waxy cuticular layer and rigid cell walls, as well as preformed antimicrobial chemicals can provide some protection against attacking phytopathogens (Nürnberger and Lipka, 2005). In addition, plants have evolved an inducible immune system that is based on the specific recognition of pathogen-derived molecules (Chisholm et al., 2006; Jones and Dangl, 2006). Two classes of plant immune receptors are critical for defense activation (Jones and Dangl, 2006; Dodds and Rathjen, 2010). Pattern recognition receptors (PRRs) directly interact with highly conserved microbe associated molecular patterns (MAMPs) activating pattern-triggered immunity (PTI; Gómez-Gómez and Boller, 2002; Zipfel et al., 2004; Segonzac and Zipfel, 2011). PTI can be attenuated or blocked by effector molecules that are secreted into plant cells by microbial pathogens that are well-adapted to their hosts (Abramovitch and Martin, 2004). The remaining weakened host immunity operating during such compatible plant/pathogen interactions [a state also referred to as effector-triggered susceptibility (ETS)] is called basal defense (Glazebrook et al., 2003; Chisholm et al., 2006; Jones and Dangl, 2006). While basal defense can limit the spread of virulent pathogens in their hosts, it is typically insufficient to prevent disease.

A second class of plant immune receptors, encoded by disease *resistance* (*R*)-genes, recognize the presence or activity of effectors and induce effector-triggered immunity (ETI), a manifestation of the well-described phenomenon of gene-for-gene resistance or race-specific resistance which leads to incompatible interactions (Flor, 1971; Nimchuk et al., 2003; Jones and Dangl, 2006; Elmore et al., 2011). ETI is a strong immune response that efficiently protects plants from avirulent pathogens and is often associated with the hypersensitive reaction (HR), a form of programmed death of plant cells at infection sites. Purified molecules or crude biochemical preparations from pathogens triggering PTI have also been referred to as general elicitors, while those triggering ETI, or race-specific resistance, have been termed race-specific elicitors (Wevelsiep et al., 1991).

Numerous studies have shown that ETI, basal defense and PTI utilize a common set of signaling components including multiple regulatory proteins, reactive oxygen intermediates (ROIs) as well as the phytohormones salicylic acid (SA), ethylene (ET) and jasmonic acid (JA; Nimchuk et al., 2003; Glazebrook, 2005; Spanu, 2012). Levels of ROI, SA, ET, or JA often increase in plant tissues after pathogen infections. While basal defense seems mainly to be a weakened form of PTI, ETI has been proposed to result from boosted basal defense- or PTI-associated responses (Tao et al., 2003; Jones and Dangl, 2006; Shen et al., 2007).

Inducible immune responses are tightly associated with extensive transcriptional- and metabolic–reprogramming controlled by a complex regulatory network (Glazebrook et al., 2003; Tsuda et al., 2009; Sato et al., 2010). While historically 10 classes of pathogenesis-related (*PR*) genes had been recognized, which exhibit transcriptional up-regulation in defense-related biological situations (Kombrink and Somssich, 1997), more recent genome-wide transcript profiling studies have revealed that 100–1000s of genes typically respond to defense induction by transiently altered transcript levels. Numerous signal transducers and transcription factors have been implicated in the plant defense network (Katagiri, 2004; Eulgem, 2005; Jones and Dangl, 2006). This network can be subdivided into various defined sectors that can interact with each other (Tsuda et al., 2009; Sato et al., 2010). For example, distinct defense signaling sectors dependent on early MAMP-activated MAP kinases (MAPKs) or the hormones SA or JA, have been described. Interestingly, some of these sectors were found to largely interact in an additive or synergistic fashion during PTI, while they are partially antagonistic to each other during ETI (Tsuda et al., 2009). The latter phenomenon seems to allow for compensatory effects if a defined sector is disabled due to interferences with pathogen effectors.

The complexity of this network is likely the result of two separate co-directional evolutionary pressures. Firstly, the asymmetrical arms race between plants and pathogens/pests manifested in continuous co-evolution of effectors and their host targets may have resulted in an ever-increasing diversity of plant defense regulators and regulatory circuits. Secondly, the need to fine-tune defense outputs appropriate for the respective attacker(s), which may exhibit biotrophic, hemibiotrophic, or nectrotrophic lifestyles, requires a complex regulatory system that allows for extensive crosstalk and compensatory interactions (Tsuda et al., 2009). An additional level of complexity likely arose from the need to link effector recognition mechanisms, which appear to be of recent evolutionary origin to more ancient regulatory processes mediating PTI (Chisholm et al., 2006; Holub, 2008).

While PTI, basal defense and ETI are transient local responses limited to pathogen infected tissues, plants can also activate long-lasting systemic immunity. Such systemic immunity can be initiated by local compatible or incompatible interactions resulting in systemic acquired resistance (SAR) or triggered by certain strains of non-pathogenic plant growth-promoting rhizobacteria (PGPR) leading to induced systemic resistance (ISR; Pieterse et al., 1998; van Wees et al., 2000). SAR mediates long-lasting broad-spectrum resistance to a wide range of pathogens in uninfected tissues and organs (Ward et al., 1991; Fu and Dong, 2013). In addition to local pathogen infections, exogenous application of SA or SA analogs (see below) can induce SAR-like responses (White, 1979; Metraux et al., 1991; Ward et al., 1991). SAR and related systemic immune responses have been demonstrated in several plant systems, such as cucumber, watermelon, tobacco, and *Arabidopsis thaliana* (*Arabidopsis*; White, 1979; Kuc, 1982; Metraux et al., 1991; Ward et al., 1991). Typically SAR is associated with a local and systemic increase of SA levels that conditions enhanced expression of several classical *PR* genes (Rasmussen et al., 1991; Ward et al., 1991; Vernooij et al., 1994; Wildermuth et al., 2001; Durrant and Dong, 2004). Some of these *PR* genes, such as *PR1*, *PR2*, and *PR5* serve as robust markers for this systemic immune response (Kombrink and Somssich, 1997).

While local and systemic accumulation of SA is critical for SAR induction, this hormone seems not to serve as a mobile signal mediating immunity in uninfected distal tissues. Several other small molecules have been proposed to fulfill such a role, such as methyl-salicylic acid (MeSA), azelaic acid, glycerol-3-phosphate, the abietane diterpenoid dehydroabietinal, JA, and the amino acid-derivative pipecolic acid (Park et al., 2007; Fu and Dong, 2013). A central regulator of SAR is the transcriptional co-factor NON- EXPRESSOR OF PR GENES1 (NPR1; Dong, 2004). By interacting with TGA bZIP transcription factors, NPR1 seems to mediate up-regulation of the vast majority of SAR-associated genes (Fu and Dong, 2013). NPR1 activity has been proposed to be controlled by the SA-binding proteins NPR3 and NPR4, which can physically bind to NPR1 in a SA-concentration-dependent manner (Fu et al., 2012).

In contrast to SAR, induction of ISR is not associated with the accumulation of SA and *PR* transcripts (Sticher et al., 1997; van Wees et al., 2000). ISR has been shown to be triggered by the *Pseudomonas fluorescens* strain WCS417r (WCS417r) and other non-pathogenic rhizobacteria in several plant species including *Arabidopsis* (Wei et al., 1996; Sticher et al., 1997; Pieterse et al., 1998; Yan et al., 2002; Vallad and Goodman, 2004). In *Arabidopsis*, WCS417r-induced ISR acts against *P. syringae* pv. tomato, is dependent on JA and ET signaling, but does not require SA. Intriguingly, ISR is blocked in the *Arabidopsis npr1* mutant. Thus, *NPR1* also plays an important role in the ISR signaling pathway (Pieterse et al., 1998; Glazebrook, 2001).

Upon perception of several exogenous defense-related stimuli, plants can establish an enhanced capacity to activate immune responses. This sensitization process, which is called priming, can be triggered by treatment of plants with necrotizing pathogens, beneficial microorganisms, wounding or with various natural and synthetic compounds (Conrath et al., 2002, 2006; Conrath, 2006; Beckers and Conrath, 2007; Goellner and Conrath, 2008). Once a pathogen infects primed plants, defense responses are activated faster and more robustly (Conrath et al., 2006; Goellner and Conrath, 2008). Although this phenomenon has been known for years, its molecular basis is still only partly understood (Conrath, 2006, 2011; Conrath et al., 2006). Chromatin modifications, accumulation of dormant mitogen-activated protein kinases and alterations of primary metabolism have been shown to be associated with this process (Conrath et al., 2002, 2006; Beckers et al., 2009; Conrath, 2011; Jaskiewicz et al., 2011).

### A BRIEF HISTORY OF SYNTHETIC ELICITORS

Synthetic elicitors are small molecules that can induce plant immune responses and are structurally distinct from natural plant defense inducers, such as general or race-specific elicitors or endogenous plant defense signaling molecules. Synthetic elicitors may trigger defense reactions by mimicking interactions of natural elicitors or defense signaling molecules with their respective cognate plant receptors or by interfering with other defense signaling components. Often the term “plant activators” is used for molecules that can protect plants from diseases by inducing immune responses. However, this term does not discriminate between synthetic and natural elicitors. One of the first synthetic elicitors was identified by Gianinazzi and Kassanis (1974), who found Polyacrylic acid derivatives of 3500 Da or lower molecular weights to mediate resistance of tobacco (*Nicotiana tabacum*) against tobacco mosaic virus (TMV) or tobacco necrosis virus (TNV) and to activate *PR1* gene expressions in tobacco (Gianinazzi and Kassanis, 1974; Kassanis and White, 1975). At the same time, 2,2-dichloro-3,3-dimethylcyclopropane-carboxylic acid (WL28325) was described as a compound suitable for controlling rice blast in rice. WL28325 affects the phenol metabolism of rice plants by enhancing peroxidase activities (Langcake and Wickins, 1975a,b). Two years later, 3-allyloxy-1,2-benzisothiazole-1,1-dioxide, widely called Probenazole (PBZ), was described. It activates defense-related enzymes and triggers dramatic increases of tolerance against rice blast in rice. It has effectively been used in agriculture for over three decades against rice blast (Watanabe et al., 1977; Schreiber and Desveaux, 2008).

Exogenous application of SA and other benzoic acid derivatives, such as acetylsalicylic acid (Aspirin), was reported to induce resistance of tobacco against TMV and to cause the accumulation of PR-proteins (White, 1979). This discovery was a major breakthrough and paved the way for the identification of more potent related compounds by the Switzerland-based pharmaceutical corporation Ciba-Geigy (now Syngenta). Ciba-Geigy researchers reported 2,6-dichloro-isonicotinic acid (INA) and its ester derivative CGA 41397 as potent SAR-inducers in 1987. They also identified benzo(1,2,3)thiadiazole-7-carbothioic acid *S*-methyl ester (BTH), which has similar effects as INA, but was later found to be more suitable for applications in crop protection (Metraux et al., 1990; Ward et al., 1991; Friedrich et al., 1996; Görlach et al., 1996; Lawton et al., 1996; Uknes et al., 1996). As INA and BTH mimic the defense-associated effects of SA, but are less phytotoxic and more efficient than this natural plant defense hormone, they have been abundantly used as defense triggers in basic and applied studies on plant immunity. As outlined in detail below, these two compounds have been among the most frequently used synthetic elicitors in research for the past 15–20 years. However, recent improvements in combinatorial chemistry (Blackwell and Zhao, 2003; Stockwell, 2004; Dean, 2005; Raikhel and Pirrung, 2005) have enabled scientists outside the private sector to perform systematic screens for synthetic elicitors. Thus, a plethora of new compounds with defense-inducing properties distinct from INA and BTH or other established synthetic elicitors is currently emerging (**Table 1**). Such second-generation synthetic elicitors will equip researchers with an extensive repertoire of new chemical tools to dissect the plant defense network in an unprecedented fashion and to explore their use as active ingredients of novel types of pesticide alternatives and other agrochemicals.

**Table 1:** Synthetic elicitors discussed in the main text.

Chemical names	Chemical structures	Biotic interactions^∗^	Application methods	Concentrations^∗∗^	Reference
3-allyloxy-1,2-benzisothiazole-1,1-dioxide(Probenazole, PBZ)	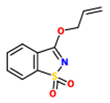	*Oryza sativa* – *Magnaporthe grisea*	Root drench	896 uM (200 ppm)	Watanabe et al. (1977)

2,6-dichloro-isonicotinic acid (INA)	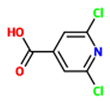	Cucumber (*Cucumis sativus*) – *Colletotrichum lagenarium*	Foliar spray	104 uM (20 ppm)	Metraux et al. (1991); Ward et al. (1991), Uknes et al. (1992)
		*Nicotiana tabacum* -*Tobacco mosaic virus* (TMV)	injection into leaves	1000 uM	
		*Arabidopsis thaliana* (Ler) *– Hyaloperonospora arabidopsidis*	Soil drench	52 uM	
		*A. thaliana* (Col-0) – *Pseudomonas syringae* pv ’tomato’ DC3000	Foliar spray	650 uM	

benzo(1,2,3)thiadiazole-7-carbothioic acid S-methyl ester (BTH)	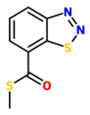	*A. thaliana* (Col-0) – *P. syringae* pv ’tomato’ DC3000	Foliar spray	300 uM	Friedrich et al. (1996), Görlach et al. (1996), Lawton et al. (1996)
		*A. thaliana* (Col-0) – *H. arabidopsidis*	Foliar spray	300 uM	
		*A. thaliana* (Col-0) – *Turnip crinkle virus*	Foliar spray	300 uM	
		*N. tabacum* – *Cercospora nicotianae*	Foliar spray	1200 uM	
		*N. tabacum – Erwinia carotovora*	Foliar spray	1200 uM	
		*N. tabacum* – *Phytophthora parasitica*	Foliar spray	1200 uM	
		*N. tabacum* – *P. syringae* pv. Tabaci	Foliar spray	1200 uM	
		*N. tabacum –* TMV	Foliar spray	1200 uM	

N-(3-chloro-4-methylphenyl)-4-methyl-1,2,3-thiadiazole-5-carboxamide (Tiadinil, TDL)	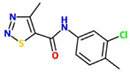	*N. tabacum* –TMV	Root drench	1 mg/pot	Yasuda et al. (2004)

Isotianil	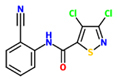	*O. sativa* – *M. grisea*	Foliar spray	840 uM (250 ppm)	Ogava et al. (2011)

*N*-*cyanomethyl-2-chloroisonicotinamide (NCI)*	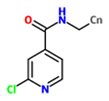	*O. sativa* – *Pyricularia oryzae*	Root drench	240 g a.i (active ingredient)/10a (are)	Yoshida et al. (1990a)

					
3-chloro-1-methyl-1H-pyrazole-5-carboxylic acid (CMPA)	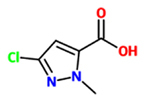	*O. sativa* – *P. oryzae*	Root drench	0.05 mg/pot	Nishioka et al. (2003, 2005)

3,5-dichloroanthranilic acid (DCA)	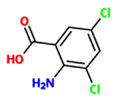	*A. thaliana* – *H. arabidopsidis*	Foliar spray	100 uM	Knoth et al. (2009)

2-[(E)-2-(2-bromo-4-hydroxy-5-methoxyphenyl)ethenyl] quinolin-8-ol) (Imprimatin A1)	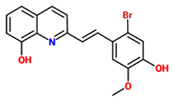	*A. thaliana–P. syringae pv tomato DC3000 avrRpm 1* or *A. thaliana–P. syringae pv. tomato DC3000*	Root drench	100 uM	Noutoshi et al. (2012d,e,f)

7-chloro-2-[(E)-2- (4-nitrophenyl)ethenyl]-4H-3,1-benzoxazin-4-one) (Imprimatin A2)	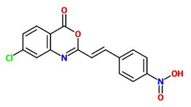	*A. thaliana* – *P. syringae* pv tomato DC3000 avrRpm 1or *A. thaliana* – *P. syringae* pv. tomato DC3000	Root drench	100 uM	Noutoshi et al. (2012d,e,f)

4-[(E)-2-(quinolin-2-yl)ethenyl]phenol) (Imprimatin A3)	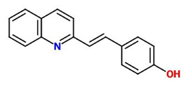	*A. thaliana* – *P. syringae* pv tomato DC3000 avrRpm 1 or *A. thaliana* – *P. syringae* pv. tomato DC3000	Root drench	100 uM	Noutoshi et al. (2012d,e,f)

2-(3-(2-furyl)-3-phenylpropyl) benzo[c]azoline-1,3-dione) (Imprimatin B1)	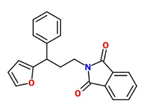	*A. thaliana* – *P. syringae* pv tomato DC3000 avrRpm 1 or *A. thaliana* – *P. syringae* pv. tomato DC3000	Root drench	100 uM	Noutoshi et al. (2012d,e,f)

3-(2-furyl)-3-phenylpropylamine) (Imprimatin B2)	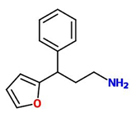	*A. thaliana* – *P. syringae* pv tomato DC3000 avrRpm 1 or *A. thaliana – P. syringae* pv. tomato DC3000	Root drench	100 uM	Noutoshi et al. (2012d,e,f)

[(E)-[1-amino-2-(2-oxopyrrolidin-1-yl)ethylidene]amino] 4-chlorobenzoate) (Imprimatin C1)	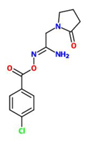	*A. thaliana* – *P. syringae* pv tomato DC3000 avrRpm 1 or *A. thaliana* – *P. syringae* pv. Tomato DC3000	Root drench	100 uM	Noutoshi et al. (2012c)

[(E)-[1-amino-2-(2-oxopyrrolidin-1-yl)ethylidene]amino]3,4-dichlorobenzoate) (Imprimatin C2)	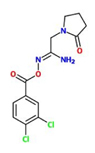	*A. thaliana* – *P. syringae* pv tomato DC3000 avrRpm 1 or *A. thaliana – P. syringae* pv. tomato DC3000	Root drench	100 uM	Noutoshi et al. (2012c)

Sulfamethoxazole (Smex)	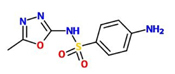	A. thaliana – P. syringae pv. tomato DC3000	Foliar spray	100 uM	Schreiber et al. (2008)

3-(butylamino)-4-phenoxy-5-sulfamoylbenzoic acid (Bumetanide)	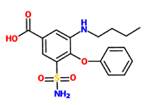	*A. thaliana* – *P. syringae* pv. tomato DC3000 (Pst) avrRpm 1	Root drench	100 uM	Noutoshi et al. (2012b)

3-benzyl-1,1-dioxo-6-(trifluoromethyl)-3,4-dihydro-2*H*-1,2,4-benzothiadiazine-7-sulfonamide (Bendroflumethiazide)	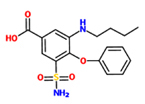	*A. thaliana* – *P. syringae* pv. tomato DC3000 (Pst) avrRpm 1	Root drench	100 uM	Noutoshi et al. (2012b)

4-chloro-*N*-(2,6-dimethyl-1-piperidyl)-3-sulfamoyl-benzamide (Clopamide)	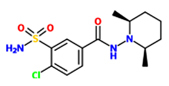	*A. thaliana* – *P. syringae* pv. tomato DC3000 (Pst) avrRpm 1	Root drench	100 uM	Noutoshi et al. (2012b)

1-oxo-indanoyl-L-isoleucine methyl ester	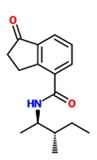	*Pennisetum glaucum – Sclerospora graminicola*	Seeds are soaked with chemical	75 uM	Deepak et al. (2007)

*^∗^Biotic interactions tested and affected by compound; ^∗∗^Converted to molarity if information on concentration available*.

## FUNCTIONAL ANALOGS OF SALICYLIC ACID

The natural plant defense hormone SA (2-hydroxybenzoic acid) serves as an endogenous signal to activate certain immune responses and to establish disease resistance. Various defense-related stimuli have been shown to trigger enhanced SA levels in local and systemic plant tissues. Exogenous application of SA can induce ROI production, *PR* gene expression and immunity against various pathogens with biotrophic or hemibiotrophic lifestyles (Glazebrook, 2005; Vlot et al., 2009).

In plants, SA can be synthesized from the shikimate pathway-derived primary metabolite chorismate either via phenlypropanoid derivatives in the cytoplasm or via isochorismic acid in chloroplasts (Pieterse et al., 2012; An and Mou, 2014). Although both metabolic pathways are not fully understood, several of their enzymes have been identified. The production of SA and its levels are normally tightly regulated (Wildermuth, 2006). Critical for the production of the majority of defense-associated SA in *Arabidopsis* is isochorismate synthase 1 (ICS1), which is transcriptionally induced by defense-related stimuli (Wildermuth et al., 2001). Two distinct forms of SA glucosyltransferase (SAGT) enzymes convert most of the produced SA to either salicyloyl glucose ester (SGE) or SA-O-β-glucoside (SAG), which is stored in the vacuole. Additional SA derivatives are known in plants, such as MeSA. SAG, SGE, and MeSA are likely biologically inactive (Vlot et al., 2009; Fu and Dong, 2013).

Salicylic acid plays a pivotal role in defense signaling and several proteins have been proposed to bind to SA and to potentially serve as SA receptors. The first putative SA-binding protein reported in the literature was SABP1 from tobacco, a potential catalase (Chen et al., 1993). It was proposed that SA inhibits its ability to convert H_2_O_2_ to O_2_ and H_2_O (Conrath et al., 1995; Du and Klessig, 1997; Vlot et al., 2009). However, this claim is controversial, as much higher SA-concentrations seem to be needed for catalase inhibition than observed in defense-activated plants (Chamnongpol et al., 1996; Tenhaken and Rubel, 1997). Similarly, it was shown that SA can also bind to ascorbate peroxidase (APX) and inhibit its activity upon application of high concentrations of exogenous SA (Durner and Klessig, 1995; Vlot et al., 2009). An additional tobacco SA-binding protein, SABP2, functions as a MeSA esterase. SABP2 shows a high binding affinity for SA, which inhibits its esterase activity (Kumar and Klessig, 2003; Forouhar et al., 2005). SABP2 seems to play an important role in the activation of SAR in tobacco by catalyzing the release of SA from the transport metabolite MeSA in systemic tissues (Park et al., 2007). Another SA-binding protein, SABP3, a tobacco chloroplastic carbonic anhydrase, is involved in HR and has antioxidant function (Slaymaker et al., 2002; Vlot et al., 2009). However, it remains to be determined whether this function can affect plant defense.

In *Arabidopsis*, NPR1 plays a critical role in the interpretation of the SA signal. NPR1 is responsible for activating a large set of defense genes in response to SA-related signals (Dong, 2004; Fu and Dong, 2013). Moreover, the NPR1 paralogues NPR3 and NPR4 function as SA receptors, and their interactions with NPR1 are directly regulated by binding to SA (Fu et al., 2012). In addition, NPR1 itself has also been shown to be capable of binding SA independently of NPR3 and NPR4 and to respond to interactions with this ligand by conformational changes (Wu et al., 2012).

With several proteins capable of binding to SA, defense mechanisms controlled by this phytohormone feature a set of “drug-able” targets potentially interfering with SA-related synthetic molecules.

Consequently, some synthetic elicitors have been found to mimic a subset of known SA functions; likely by directly interfering with known or unknown receptors of this defense hormone. Besides such SA agonists, which molecularly mimic SA, other synthetic elicitors may trigger transcriptional and physiological responses related to those induced by SA without directly interfering with SA targets. For this review we consider both types of SA mimics as functional SA analogs. Synthetic elicitors of this type are described in the section, below.

### PROBENAZOLE (PBZ)

Several biologically active 1,2-benzisothiazole derivatives have been found to exhibit a broad spectrum of pharmacological activities and to serve as antibacterials, fungicides and anti-inflammatory agents (De, 1981; Trapani et al., 1985; Zani et al., 1996; Vicini et al., 2002). Some of them also show auxin-like activity and have been used as herbicides (Giannella et al., 1971; Branca et al., 1975). Inspired by the potency of some of these compounds, researchers of Meiji Seika Kaisha Ltd. in Japan performed systematic tests with representatives of this class of molecules (Watanabe et al., 1977). They found 3-allyloxy-1,2-benzisothiazole-1,1-dioxide (now widely known as PBZ), to efficiently control rice blast (*Magnaporthe oryzae*; anamorph: *Pyricularia oryzae*) infections in rice (*Oryza sativa*; Watanabe et al., 1977; Schreiber and Desveaux, 2008). This compound showed remarkable effects in suppressing rice blast at a dose of 896 uM (200 ppm) when applied by drenching roots (Watanabe et al., 1977) and has been commercially used under the name Oryzemate^®^ for more than 30 years in the field protecting rice from rice blast fungus and bacterial leaf blight as well as corn from southern corn leaf blight (Iwata, 2001; Oostendorp et al., 2001). PBZ does not influence the growth of various tested crops, such as tomato, cucumber, Chinese cabbage, kidney bean, or rice, when sprayed at a concentration of 2240 uM (500 ppm), but at 4480 uM (1000 ppm) some abnormalities in plant development can be observed (Watanabe et al., 1977).

Probenazole affects various stages of the blast fungus infection cycle and inhibits hyphal penetration into the host tissue, lesion expansion and sporulation (Watanabe et al., 1977). From PBZ-treated rice plants anticonidial germination substances were isolated and characterized as toxic against fungi. These antifungal plant metabolites included a mixture of fatty acids, such as octadecatrienoic acid, palmitic acid, linoleic acid, and linolenic acid (Sekizawa et al., 1981; Shimura et al., 1983). Moreover, activities of defense-related enzymes, such as peroxidase, polyphenoloxidase, PAL, tyrosine ammonia-lyase and catechol-O-methyltransferase, increased dramatically in rice upon treatment with PBZ, as they do in response to infection with rice blast fungus (Midoh and Iwata, 1996; Iwata, 2001).

A PBZ-induced cDNA termed *PBZ-responsive gene* (*PBZ1*) has been cloned from rice. PBZ1 transcript accumulation was found to serve as a robust marker for responses to this synthetic elicitor. PBZ-induced PBZ1 mRNA accumulates in a dose-dependent manner. *PBZ1* expression is also induced by rice blast fungus, but not wounding. *PBZ1* belongs to the *PR-10* family of classical *PR* genes. One of the metabolites of PBZ, 1,2-benzisothiazole-3(2H)-one-1,1-dioxide (BIT) was found to be as potent in inhibiting rice blast as PBZ, but does not induce the accumulation of the PBZ1 transcripts (Midoh and Iwata, 1996; Nakashita et al., 2001, 2002b; Yoshioka et al., 2001). Thus, induced *PBZ1* expression seems not to be needed for rice blast resistance.

Microarray and RT-PCR analysis revealed up-regulation of UDP-glucose:SA glucosyltransferase (*Os*SGT1) transcripts in response to PBZ treatment in rice (Umemura et al., 2009). RNAi-mediated *Os*SGT1 knockdown in transgenic rice plants resulted in reduced PBZ-mediated resistance against blast. Although mechanistic details of its role in defense induction are unclear, *Os*SGT1 appears to be critical for PBZ-mediated defense induction (Umemura et al., 2009).

In *Arabidopsis*, both PBZ and its metabolite BIT stimulate expression of *PR* genes and induce SA accumulation and SAR. PBZ and BIT do not activate plant immunity in *npr1* mutants or *nahG* plants. Thus, SA and NPR1 seem to be required for PBZ- and BIT-mediated defense responses and both compounds mimic effects of SA (Yoshioka et al., 2001; Nakashita et al., 2002b). However, in contrast to INA, BTH, and DCA, which are likely authentic SA agonists (see below), PBZ and BIT appear to interfere with defense signaling steps upstream from SA accumulation and not to interact with downstream targets of SA.

### 2,6-DICHLORO-ISONICOTINIC ACID (INA)

Kunz et al. (1988) of Ciba-Geigy reported screening of a large number of compounds for activation of resistance in cucumber (*Cucumis sativus*) against the fungal pathogen *Colletotrichum lagenarium* and identified 2,6-dichloro-isonicotinic acid (INA) and its ester derivative CGA41397 (Kunz et al., 1988; Metraux et al., 1991). High levels of protection of cucumber against *C. lagenarium*, were achieved by foliar-spray application of 104 uM (20 ppm) INA or CGA41397 as well as root drench application of 10-fold lower concentrations of each compound. In these chemically-treated plants, responses were similar to those observed in systemic tissues of plants whose lower leaves were inoculated with TNV *or C. lagenarium* that induce SAR in upper leaves. Under field conditions, INA provided pathogen resistance in pear, pepper and rice (Kuc, 1982; Metraux et al., 1991). INA was also shown to induce SAR in tobacco and *Arabidopsis (*Ward et al., 1991; Uknes et al., 1992*)* and provide significant protection of tobacco against TMV, *Cercospora nicotianae, Peronospora tabacina, Phytophthora parasitica* var nicotianae, and *P. syringae* pv. tabaci (Ward et al., 1991).

In *Arabidopsis* INA can trigger long-lasting *PR* gene expression and disease resistance. In this species it can reduce susceptibility to virulent strains of the oomycete *Hyaloperonospora arabidopsidis (Hpa*) or *P. syringae* pv. tomato DC3000 without directly affecting viability of these pathogens (Uknes et al., 1992; Knoth et al., 2009). As injection of 1 mM INA into tobacco leaves induces transcript accumulation of the same characteristic set of *PR* genes as SA application, it is considered a functional SA analog. Although INA partially mimics defense-associated effects of SA, it does not trigger any changes of SA levels and, unlike SA or PBZ, induces SAR in *nahG* transgenic tobacco and *Arabidopsis* plants (Delaney et al., 1994; Vernooij et al., 1995). Thus, INA must be interfering with targets that operate downstream from SA accumulation and are likely involved in the interpretation of SA levels. Consistent with this assumption, INA has been reported to mimic several proposed biochemical and physiological effects of SA, such as inhibition of catalase and APX activity or the induction of cellular H_2_O_2_ accumulation (Chen and Klessig, 1991; Chen et al., 1993, 1995; Conrath et al., 1995; Durner and Klessig, 1995). The modulation of ROI levels seems to be a critical aspect of INA activity, since antioxidants can block the INA-dependent induction of *PR* gene expression (Chen et al., 1995; Durner and Klessig, 1995).

Through mutant screens to identify genes required for SAR in *Arabidopsis*, the *npr1/nim1* (*non-expresser of PR genes 1, no immunity 1*) mutants that are insensitive to SA and INA were discovered (Cao et al., 1994; Delaney et al., 1995). Both biologically- and INA-induced SAR as well as basal defense were found to be compromised in either one of these mutants. The *npr1* and *nim1* mutants are in different *Arabidopsis* accessions, but were found to be allelic and to have defects in the same gene (Cao et al., 1994, 1997; Ryals et al., 1997). A large body of literature has reported on molecular roles of NPR1 as a transcriptional cofactor, since its identification as a major regulator of SAR. These studies have been summarized in several excellent reviews (Dong, 2004; Durrant and Dong, 2004; Fu and Dong, 2013). Most importantly, NPR1, together with NPR3 or NPR4, have been found to serve as SA receptors (Fu et al., 2012; Fu and Dong, 2013). NPR3 can bind to NPR1 in a SA dose-dependent manner, while NPR4-NPR1 interactions are constitutive and inhibited by SA. In yeast two-hybrid assays, in addition to SA, INA can promote NPR1–NPR3 interactions. INA can also reduce the binding affinity of SA to NPR3 and NPR4 by competing with this defense hormone (Fu et al., 2012). Thus, INA appears to be a true SA agonist.

In addition to *npr1* mutants, triple or quadruple mutants of closely related TGA-bZIP transcription factors, which are known to physically interact with NPR1, are also blocked in INA-induced *PR* gene expression and pathogen resistance (Zhang et al., 2003; Wang et al., 2006). Thus, INA seems to mediate its defense-related effects upon interactions with NPR1-related proteins, which control several TGA transcription factors. Interactions with other SA-binding proteins, such as SABP1 and SABP2 may also to contribute to the activity of this SA analog. So far, INA has been applied to many plant species and was found to induce resistance against a wide variety of pathogens (Hijwegen and Verhaar, 1993; Conrath et al., 1995; Van Kan et al., 1995; Han et al., 2000; Lee et al., 2009). However, because INA and its derivatives have phytotoxic side effects in crops, none of these compounds has been commercialized as agrochemicals (Oostendorp et al., 2001). Still, INA is being continually used as an efficient chemical tool to study SAR.

### BENZOTHIADIAZOLE (BTH)

Another SAR-inducer screening by Ciba-Geigy with a large number of benzo[1,2,3]thiadiazole-7-carboxylic acid derivatives resulted in the identification of benzo(1,2,3)-thiadiazole-7-carbothioic acid S-methyl-ester [benzothiadiazole (BTH); acibenzolar-S- methyl (ASM), CGA245704] as a potent inducer of plant immune responses (Schurter et al., 1993; Kunz et al., 1997; Oostendorp et al., 2001). BTH was subsequently shown to trigger in various plant species resistance against a wide variety of pathogens, such as TMV, *Cercospora nicotianae, Erwinia carotovora, Phytophthora parasitica* and *P. syringae* pv. tabaci (Friedrich et al., 1996; Görlach et al., 1996; Lawton et al., 1996; Kunz et al., 1997). As BTH did not show any direct effect on a number of plant pathogens *in vitro*, BTH is not antimicrobial (Friedrich et al., 1996). In *Arabidopsis*, BTH triggers *NPR1*-dependent SAR (Lawton et al., 1996).

At the molecular level, BTH induces the same characteristic set of SAR-related responses that are induced by pathogens or SA, including up-regulation of *PR* genes. Thus, like INA, BTH appears to be a functional analog of SA (Friedrich et al., 1996; Wendehenne et al., 1998). INA and BTH share several characteristic functional features. Both compounds do not induce accumulation of SA in plants (Vernooij et al., 1995; Friedrich et al., 1996) and share the ability to induce SAR and *PR* gene expression in transgenic *nahG* lines (Vernooij et al., 1995; Lawton et al., 1996). Thus, both INA and BTH seem to activate SA-response mechanisms by interfering as SA agonists with targets operating downstream from SA accumulation. Like SA and INA, BTH was also proposed to inhibit both APX and catalase functions (Du and Klessig, 1997; Wendehenne et al., 1998). However, BTH is a much more effective inhibitor of catalase than SA and the catalase inhibition mechanisms of BTH and SA are different. While SA seems to inhibit catalase function in an H_2_O_2_- and time-dependent manner, BTH inhibits this activity independently from time and H_2_O_2_. INA was not included in these experiments_._ For APX inhibition, however, BTH and SA exhibit similar dose-response curves (Wendehenne et al., 1998).

Recent data suggested that BTH is converted into acibenzolar by SABP2 and this product is critical for SAR induction. When BTH was sprayed on SABP2-silenced tobacco plants, it failed to induce PR1 protein expression and SAR. On the contrary, when the same transgenic plants were treated with acibenzolar, SAR was fully induced (Tripathi et al., 2010).

In rice, it was shown that the *Os*WRKY45 transcription factor plays a pivotal role in BTH-induced defense responses against rice blast disease. This BTH-triggered defense mechanism seems independent of NH1, a rice ortholog of *A. thaliana* NPR1 (Shimono et al., 2007). WRKY45 knockdown lines exhibited strongly reduced levels of BTH-induced resistance to the fungal pathogen *M. oryzae* and the bacterial pathogen *Xanthomonas oryzae* pv. oryzae (Xoo; Shimono et al., 2007). Interestingly, *Os*WRKY45 is an ortholog of *At*WRKY70, which also can act in an NPR1-independent manner in SA signaling in *Arabidopsis* (Li et al., 2004; Knoth et al., 2007, 2009). In addition to BTH, PBZ and Tiadinil (TDL; see below) partly induced blast resistance in rice through a WRKY45-dependent pathway (Shimono et al., 2012). Recently, WRKY45-regulated BTH-responsive genes were identified by microarrays (Nakayama et al., 2013).

BTH can also prime plant defense reactions. Low doses of BTH that are insufficient to trigger detectable levels of defense responses, can prime parsley cells and increase their sensitivity for MAMP-triggered coumarin phytoalexin secretion. This effect is associated with potentiated activation of genes encoding phenylalanine ammonia-lyase (PAL), which is critical for coumarin biosynthesis. In addition to BTH, also SA and INA can prime parsley cells for the activation of coumarin secretion by low MAMP doses (Kauss et al., 1992; Katz et al., 1998; Thulke and Conrath, 1998; Conrath et al., 2002). BTH can also prime *Arabidopsis* plants for enhanced pathogen-responsiveness of *PAL* gene expression. BTH-mediated defense priming in *Arabidopsis* is dependent on NPR1 (Kohler et al., 2002; Goellner and Conrath, 2008). An interesting mechanism involving two known defense-associated MAPKs, MPK3, and MPK6, seems to contribute to this priming phenomenon in *Arabidopsis*. BTH induces the accumulation of mRNA and inactive protein forms of both MAPKs. Subsequent stress treatment results in phosphorylation and activation of MPK3 and MPK6 (Beckers et al., 2009). In addition, epigenetic chromatin marks appear to be involved in defense-priming processes. The *AtWRKY29*, *AtWRKY6,* and *AtWRKY53* genes showed a typical priming response and were strongly transcribed after stress application following pre-treatment with BTH. BTH pre-treatment also triggered in these experiments various histone modifications that are typically found at actively transcribed genes, such as H3K4me3, H3K4me2, H3ac, or H4ac at *AtWRKY29* and H3K4me3 or H3K4me2 at *AtWRKY6* and *AtWRKY53*. BTH-induced trimethylation of H3K4 is reduced in the priming-deficient *npr1* mutant. On the contrary, the constitutively primed *cpr1* and *sni1* mutants exhibit high levels of H3K4me3 in the absence of BTH treatment. Thus, elevated H3K4me3 levels are closely associated with BTH-induced defense gene priming (Jaskiewicz et al., 2011).

In contrast to INA, BTH was found to be suitable for agricultural crop protection. It became a commercial product under the trade name of BION^®^ (in Europe) in 1989 and Actigard^®^ (in the US) in 1990 (Schurter et al., 1993; Kunz et al., 1997; Oostendorp et al., 2001). BTH activates very wide spectrum of resistances of various plant species against fungal, bacterial, or viral pathogens and several insects and nematodes.

### *N*-(3-CHLORO-4-METHYLPHENYL)-4-METHYL-1,2,3-THIADIAZOLE-5-CARBOXAMIDE (TIADINIL, TDL)

Thiadiazoles are known to have many pharmacological activities (Camoutsis et al., 2010; Chaudhary et al., 2010; Kharb et al., 2011; Singh et al., 2011). Tests of various 1,2,3-thiadiazole derivatives for their ability to control rice blast disease by Nihon Nohyaku Co., Ltd. (Japan) resulted in the discovery of *N*-(3-chloro-4-methylphenyl)-4-methyl-1,2,3-thiadiazole-5-carboxamide (Tiadinil, TDL), which provided protection against this disease without exhibiting any antimicrobial activity (Tsubata et al., 2006). Since 2003, this compound has been commercially available under the trade name V-GET^®^in Japan. Its metabolite 4-methyl-1,2,3-thiadiazole-5-carboxylic acid (SV-03), exhibited similar levels of anti-rice blast activity as TDL (Tsubata et al., 2006; Toquin et al., 2012). In addition to rice blast, TDL is also used to control the pathogenic fungi *Colletotrichum theaesinensis* and *Pestalotiopsis longiseta* on tea leaves (Yoshida et al., 2010).

In tobacco, TDL and SV-03 induce SAR and increased local resistance to TMV, the virulent bacterial pathogen *P. syringae* pv. tabaci and powdery mildew (*Oidium lycopersici*) without affecting these pathogens directly. Both compounds also induce *PR1*, *PR2* and *PR5* gene expression in *Arabidopsis* and enhance basal resistance of this species to *P. syringae* pv. tomato DC3000 (Yasuda et al., 2004, 2006; Yasuda, 2007). TDL or SV-03 treatment does not induce accumulation of SA in tobacco. Moreover, TDL or SV-03-treated *nahG* transgenic tobacco plants exhibit enhanced resistance to TMV and *P. syringae* pv. tabaci and induced *PR* gene expression. However, TDL- or SV-03-triggered defense responses are blocked in *Arabidopsis npr1* mutants. Taken together, these results suggest that, similar to BTH and INA, TDL and SV-03 trigger disease resistance by interfering with signaling steps downstream of SA (Yasuda et al., 2006; Yasuda, 2007).

The thiadiazole derivative, 1,3,4-oxadiazole, has also been shown to exhibit antifungal and antibacterial activities (Kharb et al., 2011; Singh et al., 2011). By combining different heterocyclic thiadiazole-related moieties, including oxadiazoles, new compounds were designed and evaluated regarding their performance in crop disease protection. Although only three out of the 23 tested compounds elicited SAR more efficiently than TDL, combining thiazole- and oxadiazole moieties may be a promising approach in designing new crop protectants (Fan et al., 2009).

### ISOTIANIL

As a result of a comprehensive search for isothiazole-based compounds, Isotianil was discovered by Bayer AG (now Bayer CropScience AG) in Germany in 1997 and developed jointly with the Japanese company Sumitomo Chemical Co., Ltd. as a crop protectant against rice blast and bacterial leaf blight in rice. It also activates defense responses against a wide range of additional pathogens in various plants. Moreover, Isotianil does not show any direct antimicrobial activity against bacteria and fungi (Ogava et al., 2011; Toquin et al., 2012). In 2010, it was registered under the name Stout^®^ in Japan and China, where it substantially increased rice production (Ogava et al., 2011; Brozek et al., 2012; Yoshida et al., 2013). Its efficiency against rice blast seems unusually high, as lower dosages of Isotianil are needed than of any other existing plant defense activator, such as PBZ and TDL (Ogava et al., 2011).

At the molecular level, Isotianil treatment triggers accumulation of defense-related enzymes such as lipoxygenase or PAL in rice. Affymetrix whole genome microarray analysis revealed that Isotianil treatment induces some defense-related genes, including *OsWRKY45,* that are involved in SA signaling (Ogava et al., 2011; Toquin et al., 2012). Further microarray analyses showed that Isotianil likely primes rice for more intense defense activation in response to pathogen infections. At this point no published information on its mode-of-action is available.

### *N*-CYANOMETHYL-2-CHLOROISONICOTINAMIDE (NCI)

A screen of 2-chloroisonicotinamide derivatives for effective rice blast control agents were performed by Nihon Nohyaku Co., Ltd. (Japan), resulted in the identification of *N*-cyanomethyl-2-chloroisonicotinamide (NCI) as a potent defense inducer (Yoshida et al., 1989, 1990a,b). NCI showed one of the highest anti-blast activities compared to other *N*-alkyl-2-chloroisonicotinamides and its efficacy was equal to that of PBZ. It does not show antifungal activity against rice blast *in vitro* at concentrations as high as 1100 uM (500 ppm). Its activity is long-lasting, as it was found to be still effective against rice blast 30 days after a single application. NCI treatment inhibits mycelial development of *P. oryzae* at inner epidermal cells and increases the number of small brownish lesions that are correlated with active immunity of rice. These results suggest that NCI efficiently induces plant defense mechanisms (Yoshida et al., 1990a).

In tobacco, NCI can induce SAR and mediate local resistance to TMV, *Oidium lycopersici* and *P. syringae* pv. tabaci. It also induces expression of *PR1*, *PR2* and *PR5* and is active in transgenic *nahG* tobacco plants. Thus, it does not require SA for activation of defense (Nakashita et al., 2002a). In *Arabidopsis*, NCI reduces growth of virulent *P. syringae* and induces resistance independently from SA accumulation, ET and JA, but requires NPR1. Thus, like INA and BTH, NCI seems to interfere with defense signaling steps operating between SA and NPR1 (Yasuda et al., 2003a; Yasuda, 2007).

### 3-CHLORO-1-METHYL-1H-PYRAZOLE-5-CARBOXYLIC ACID (CMPA)

A screen by Nishioka et al. (2003) targeting new chemicals to control blast disease in rice resulted in the discovery of pyrazolecarboxylic acid derivatives as potent inducers of systemic immunity. The most efficient anti-blast compound identified in this screen was 3-chloro-1-methyl-1H-pyrazole-5-carboxylic acid (CMPA). CMPA does not directly affect pathogen viability up to a concentration of 623 uM (100 ppm), while it can significantly induce rice blast resistance at 10-fold lower concentrations. Thus, its anti-blast activity is not dependent on antimicrobial activity and this compound seems to activate systemic plant defense mechanisms (Nishioka et al., 2003). Although, CMPA, BTH, and PBZ trigger rice blast resistance with similar efficacies, CMPA induces PBZ1 transcript accumulation in rice at levels lower than PBZ or BTH (Nishioka et al., 2005).

In tobacco, CMPA enhances resistance to *P. syringae* pv. tabaci and *Oidium sp*.. CMPA also induces expression of *PR1*, *PR2,* and *PR5* in wild-type as well as *nahG* transgenic tobacco. Therefore, CMPA seems not to require SA to induce SAR-like disease resistance and may interfere with defense signaling downstream from SA. Consistent with this assumption, CMPA was found to act through NPR1 in *Arabidopsis* (Yasuda et al., 2003b; Yasuda, 2007).

### 3,5-DICHLOROANTHRANILIC ACID (DCA)

The compound 3,5-dichloroanthranilic acid (DCA) is one of 114 synthetic elicitor candidates that were identified by a comprehensive screening of 60,000 diverse compounds for inducers of the pathogen-responsive *CaBP22::GUS* reporter gene in *Arabidopsis* (Knoth et al., 2009; Knoth and Eulgem, 2014). DCA efficiently triggers resistance of *Arabidopsis* against virulent strains of the oomycete *Hpa* and *P. syringae* DC3000. It up-regulates transcript levels of various known SA-responsive defense-related genes, such as *PR1*, *WRKY70,* and *CaBP22*. Like INA and BTH, its activity does not require accumulation of SA. However, unlike these well-characterized SA analogs, DCA-mediated immunity is not fully blocked in *npr1 Arabidopsis* mutants. DCA-triggered immune responses are to a large extent independent from NPR1, but partially blocked in *wrky70* mutants. Thus DCA partially targets a WRKY70-dependent branch of the defense signaling network that does not require NPR1 (Knoth et al., 2009).

Microarray analyses revealed that DCA, INA, and BTH trigger partially overlapping transcriptional responses in *Arabidopsis* (Wang et al., 2006; Knoth et al., 2009; Bhattarai et al., 2010). For example, transcripts of a set of 202 genes were found to be commonly up-regulated by each one of these three synthetic elicitors. However, DCA, INA, and BTH also induce unique transcriptional changes. Taken together, these and other observations suggest that each of these SA analogs interferes with targets in the SA response pathway in a unique manner.

### ADDITIONAL FUNCTIONAL ANALOGS OF SA

Besides the functional analogs of SA that are discussed above, additional derivatives of this defense hormone were tested (Conrath et al., 1995; Knoth et al., 2009). This includes 3,5-dichlorosalicylic acid, 4-chlorosalicylic acid, and 5-chlorosalicylic acid, which mimic SA, induce *PR1* gene expression and enhance disease resistance to TMV infection in tobacco (Conrath et al., 1995). Furthermore, 3-chlorobenzoic acid and 3,5-dichlorobenzoic acid induce basal defense against *Hpa* as well as *CaBP22::GUS* expression in *Arabidopsis* (Knoth et al., 2009). In contrast, the SA-related compounds benzoic acid, 2-aminobenzoic acid, 3-hydroxybenzoic acid, 4-hydroxybenzoic acid, 2,3-dihydroxybenzoic acid, 2,4-dihydroxybenzoic acid, 2,5-dihydroxybenzoic acid, and 4-amino-SA did not show any defense-inducing activity (Chen and Klessig, 1991; Conrath et al., 1995; Durner and Klessig, 1995).

Furthermore, several agonists of the peroxisome proliferator-activated receptor were found to mimic effects of SA in local HR responses, but not *PR* gene expression or SAR, in soybean. The latter finding suggested that the roles of SA in local and systemic defense induction are distinct (Tenhaken et al., 2001).

## IMPRIMATINS

A screen of 10,000 small molecules to identify plant immune priming compounds by Noutoshi et al. (2012d) and coworkers resulted in the identification of three distinct classes of compounds that can prime *Arabidopsis* cells to exhibit enhanced immunity against virulent and avirulent *P. syringae*. These immune-priming compounds were termed Imprimatins. Based on structural similarities they were classified as Imprimatin A, -B or -C, representatives, respectively (**Table 2**; Noutoshi et al., 2012c,d,e,f).

**Table 2:** Imprimatins.

Main type	Common name	Systematic name
Imprimatin A	Imprimatin A_1_	2-[(E)-2-(2-bromo-4-hydroxy-5-methoxyphenyl)ethenyl] quinolin-8-ol)
	Imprimatin A_2_	7-chloro-2-[(E)-2- (4-nitrophenyl)ethenyl]-4H-3,1-benzoxazin-4-one)
	Imprimatin A_3_	4-[(E)-2-(quinolin-2-yl)ethenyl]phenol)
Imprimatin B	Imprimatin B_1_	2-(3-(2-furyl)-3-phenylpropyl) benzo[c]azoline-1,3-dione)
	Imprimatin B_2_	3-(2-furyl)-3-phenylpropylamine)
Imprimatin C	Imprimatin C_1_	[(E)-[1-amino-2-(2-oxopyrrolidin-1-yl)ethylidene]amino] 4-chlorobenzoate)
	Imprimatin C_2_	[(E)-[1-amino-2-(2-oxopyrrolidin-1-yl)ethylidene]amino]3,4-dichlorobenzoate)

A common feature of Imprimatin A and Imprimatin B compounds is that they only prime plants for enhanced defense reactions and cannot directly induce immune responses (Noutoshi et al., 2012e,f). Application of each of these compounds increases levels of endogenous SA and decreases levels of the inactive SA metabolite SAG suggesting they inhibit SAGTs (Noutoshi et al., 2012e,f). Supporting this view, single and double knockout mutants of the *Arabidopsis* SAGT genes *UGT74F1* and *UGT76B1* showed increased disease resistance and free SA levels and resemble in this respect wild-type *Arabidopsis* plants treated with Imprimatins A_1,_ A_2,_ A_3,_ B_1_, or B_2_ (Noutoshi et al., 2012e). The enzymatic activities of UGT74F1 and UGT76B1 were also blocked *in vitro* by each of these Imprimatins at concentrations effective for immune priming_._ These results suggest that Imprimatin A and -B representatives have a unique mode-of-action in defense priming and specifically inhibit SAGTs (Noutoshi et al., 2012e,f).

Two members of class C of Imprimatins, C_1_ and C_2_, were found to be SA analogs, as they activate downstream SA signaling steps and induce expression of known SA-responsive genes. However, their defense-inducing activity is weaker than that of SA. Further structure-function analyses suggested that these compounds may be converted in *Arabidopsis* to 4-chlorobenzoic acid and 3,4-chlorobenzoic acid, which can mimic the defense-related effects of Imprimatins C1 and C2 (Noutoshi et al., 2012c).

## SULFONAMIDES

### SULFANILAMIDES

In order to identify small molecules that reduce susceptibility of *Arabidopsis* to virulent *P. syringae*, a small collection of 200 molecules from the LATCA library (Library of Active Compounds in *Arabidopsis*; Zhao et al., 2007) was screened for candidates that reduce cotyledon bleaching in liquid grown seedlings. *P. syringae* induced bleaching of *Arabidopsis* cotyledons is a robust disease symptom that develops within 4–5 days post-inoculation with this pathogen (Schreiber et al., 2008). Among other candidates, the sulfanilamide compounds, sulfamethoxazole (Smex), sulfadiazine (Sdiz), and sulfapyridine (Spyr) were found to reduce this bleaching phenotype. Although, sulfanilamides have been widely used as antibiotics, the authors showed that these three candidates did not directly reduce bacterial viability and growth at concentrations that suppress their virulence. Thus, these compounds seem to act by inducing plant immune responses (Schreiber et al., 2008).

Sulfamethoxazole was found to be the most potent one of the three identified sulfanilamides. Smex can prevent cotyledon bleaching at a concentration of 100 uM. Interestingly, Smex does not induce *PR1* expression and is active in *npr1* mutants. Thus, Smex is likely to induce defense mechanisms unrelated to the canonical SA defense pathway. Smex-mediated disease protection is also independent from JA, ET, and ABA signaling and does not require an oxidative burst (Schreiber and Desveaux, 2008; Schreiber et al., 2008).

Sulfanilamides are structural analogues of *p*-aminobenzoic acid (PABA), which can inhibit dihydropteroate synthase, an enzyme that catalyzes an important step in the folate biosynthetic pathway. Smex-mediated inhibition of folate biosynthesis may induce plant defense mechanism independently from *PR1* expression (Schreiber et al., 2008, 2012). A screen performed by the same lab to identify compounds that protect *Arabidopsis* against the fungal pathogen *Fusarium graminearum* resulted, besides Smex, in the identification of the indole alkaloid gramine as a plant defense inducer. Both gramine and Smex reduced severity of *F. graminearum* infection in wheat as well (Schreiber et al., 2011).

### OTHER SULFONAMIDES

Noutoshi et al. (2012a), additional sulfonamide compounds were also reported to induce disease resistance in plants. By using the same chemical screening strategy that was used for Imprimatins, chemical libraries representing 2677 bioactive molecules and small natural compounds were screened to identify immune-priming molecules. Four different sulfonamide compounds, sulfameter (SFM), sulfamethoxypyridazine (SMP), sulfabenzamide (SBA), and sulfachloropyridazine (SCP) were identified in this screening and further characterized. They increased the occurrence of cell death of *Arabidopsis* suspension cell cultures infected by an avirulent *P. syringae* strain and were classified as immune-priming compounds. However, unlike Smex, these compounds can induce *PR1* gene expression and, unlike Imprimatin A or B representatives, they do not inhibit SAGTs (Noutoshi et al., 2012a).

### DIURETICS

Diuretics are pharmaceutical drugs that are widely used in clinical medicine, especially to treat hypertensive and oedematous states (Plant, 2003). Three diuretics, 3-(butylamino)-4-phenoxy-5-sulfamoylbenzoic acid (Bumetanide), 3-benzyl-1,1-dioxo-6-(trifluoromethyl)-3,4dihydro-2*H*-1,2,4-benzothiadiazine-7-sulfonamide (Bendroflumethiazide) and 4-chloro-*N*-(2,6-dimethyl-1-piperidyl)-3-sulfamoyl-benzamide (Clopamide; McNeil et al., 1987; Breyer and Jacobson, 1990; Pacifici, 2012) were identified as plant immune-priming compounds through the screening of a chemical library of 2000 known bioactive compounds (Noutoshi et al., 2012b). They stimulate pathogen-induced cell death in *Arabidopsis* in a concentration-dependent manner. In *Arabidopsis* they can enhance disease resistance to both avirulent and virulent *P. syringae* strains. Effects of 100 uM diuretic on defense induction are comparable to those triggered by 50 uM SA and they do not directly inhibit bacterial growth up to concentration of 200 uM. Application of these diuretics significantly decreases the growth of avirulent bacteria compared to mock treatment and mediates enhanced *PR1* gene expression after infection with *P. syringae*. These compounds potentiate disease resistance by enhancing plant defense responses, but, unlike SA and its analogs, do not induce *PR1* expression in the absence of pathogen infection (Noutoshi et al., 2012b).

Diuretics exhibit pharmacological effects in humans by acting on proteins of the SLC12A family, which are sodium-coupled chloride co-transporters that are located along the renal tubule of the kidney nephron. Diuretics inhibit these co-transporters by binding to their Cl^-^ binding site (Breyer and Jacobson, 1990; Gamba, 2005). The *Arabidopsis* genome encodes only a single protein closely related to SLC12A, *At*1g30450 (*At*CCC1). Thus, diuretics-triggered defense priming may be mediated via *At*CCC1. However, no results regarding this possible role of *At*CCC1 have been reported.

Interestingly, diuretics contain a sulfonamide moiety similar to those identified in the defense-inducing sulfanilamide compounds sulfamethoxazole, sulfadiazine, and sulfapyridine (Schreiber et al., 2008). Both diuretics and sulfanilamides can decrease bacterial growth *in planta.* The presence of sulfonamide moieties seems to be essential for their ability to induce defense reactions, as diuretics without sulfonamide groups do not exhibit this activity (Schreiber et al., 2008; Noutoshi et al., 2012b). Further studies with diuretics and sulfanilamides are needed to uncover their modes-of-action.

## ADIPIC ACID DERIVATIVES

In order to identify chemical mixtures that can delay senescence and induce immunity in plants, various mixtures of adipic acid monoethyl ester derivatives were tested. Application of a mixture of furfurylamine and 1,2,3,4-tetra-O-acetyl-β-D-glucopy-ranose (FGA) increased chlorophyll content, cell wall sugar content and delayed the chlorophyll degrading rate along with senescence in tomato and pepper (Flors et al., 2001). FGA also increased PAL activity as well as the concentration of flavonoids and phenolic compounds and strengthened plant immunity against various different pathogens such as *Phytophthora citrophthora* and *Altemaria solani* in tomato (*Solanum lycopersicum L*.) as well as *Alternaria solani* in pepper (*Capsicum annuum* L.; Flors et al., 2001). Individual application of three novel amides of adipic acid, 5-carbamoil ethyl pentanoate (N1), 5-(2-furfurylmethylcarbamoil) ethyl pentanoate (N2) and 5-(3-aminopropylcarbamoil) ethyl pentanoate (N3) was shown to strongly induce resistance against *Alternaria solani* in pepper. However, many other adipic acid derivatives were most effective when used as a mixture (Flors et al., 2003a,b). Although these chemicals reduced pathogen growth in their hosts, many of them did not show any direct antimicrobial effect to pathogens and, therefore, likely induce plant immune responses (Flors et al., 2001, 2003a,b, 2004). However, the mode-of-action underlying this function remains unresolved.

## JASMONIC ACID ANALOGS

Jasmonic acid and its methylester, methyl-jasmonate (MeJA), are important members of the family of jasmonates which are biologically active fatty-derived cyclopentanones, that are broadly present in the plant kingdom. They are synthesized rapidly by the octadecanoid (and possibly hexadecanoid) biosynthesis pathways upon pathogen or insect attack and activate defense responses (Howe, 2010; Wasternack and Hause, 2013). Jasmonates are known to control stress responses against nectrotrophic pathogens, herbivores and wounding, but are also known to perform various important roles in plant development related to leaf senescence, growth inhibition and floral development (He et al., 2002; Balbi and Devoto, 2008; Zhang and Turner, 2008; Oh et al., 2013; Santino et al., 2013). Upon synthesis, JA can either be metabolized to MeJA or conjugated to L-isoleucine leading to jasmonoyl-isoleucine (JA-Ile), which is an active form of JA (Svoboda and Boland, 2010; Pieterse et al., 2012).

Together with Jasmonate ZIM-domain (JAZ)-type transcriptional repressors, the F-box protein Coronatine Insensitive1 (COI1) functions as JA-Ile receptors. Recruitment of JAZ proteins into COI1-containing SKP1-Cullin-F-box (SCF^COI1^) complexes results in proteasome-mediated degradation of these transcriptional repressors. Consequently expression of a large number of JA-responsive genes is de-repressed and defense responses are activated (Browse, 2009; Pieterse et al., 2012; Monte et al., 2014). Jasmonates typically promote defense responses against necrotrophic microbial pathogens. For example, exogenous application of JA or MeJA was shown to protect barley against *Erysiphe graminis* f.sp. hordei (Schweizer et al., 1993). In *Arabidopsis*, MeJA up-regulates transcript levels of the *PDF1.2* gene family along with 100s of additional genes (Schenk et al., 2000; Jung et al., 2007; Scranton et al., 2013) and enhances resistance to various necrotrophic pathogens, such as the fungi *Alternaria brassicicola* and *Botrytis cinerea* (Thomma et al., 1998; Seo et al., 2001; Rowe et al., 2010).

Systematic structural modifications of JA revealed the minimal structural requirements required for its bioactivity allowing for the synthesis of JA-mimics (Svoboda and Boland, 2010). The synthetic JA mimic coronalon (2-[(6-ethyl-1-oxo-indane-4-carbonyl)-amino]-3-methyl-pentanoic acid methyl ester) mediated induction of stress responses in various plant species (Schüler et al., 2004). In addition, coronalon and its unsubstituted form (1-oxo-indanoyl-L-isoleucine methyl ester) increased levels of nicotine and trypsin proteinase inhibitors which are known MeJA-activated defense products in *N. attenuata.* They also triggered transcriptional up-regulation of the majority of genes that are known to be responsive to MeJA (Pluskota et al., 2007). The compound 1-oxo-indanoyl-L-isoleucine methyl ester was also shown to enhance activity of defense-related enzymes such as PAL or peroxidases and to induce resistance against downy mildew (Deepak et al., 2007). Additional synthetic JA mimics were shown to induce jasmonate signaling and immune responses in various plant species (Krumm et al., 1995; Fliegmann et al., 2003; Pluskota et al., 2007), However, none of these compounds were studied at the molecular level and nothing is known about their modes-of-action.

## CONCLUSIONS AND PERSPECTIVES

In this review article we have provided an overview of the discovery and functional characteristics of synthetic elicitors as well as their potential for basic research and crop protection. In our opinion, three major observations stand out.

(1) The vast majority of known synthetic elicitors belongs to the large group of functional SA analogs and mimics roles of this messenger molecule in defense induction. Many of these compounds are structurally related to SA. This strong trend may be partially due to a bias in the used compound screening strategies, most of which were based on the use of known SA-triggered immune responses as an indicator of defense induction. However, the dominance of functional SA analogs among known synthetic elicitors may also reflect that the SA-response pathway is particularly enriched for drug-able targets (which often have natural ligand binding pockets) and may involve more than just one type of SA receptor. This is consistent with the fact that responses triggered by different SA analogs do often not fully overlap and are partly unique. Thus, many functional SA analogs may constitute selective SA agonists, each of which interferes in a distinct manner with natural SA targets.
(2) Synthetic elicitors can be successfully applied in crop protection. Several examples illustrate the utility of plant immune-stimulants or -inducers in agriculture. Most likely more examples will follow, providing attractive alternatives to conventional biocidal agrochemicals.
(3) Synthetic elicitors can also serve as potent tools in basic research approaches expanding our knowledge of plant immunity. A particularly prominent example highlighting their potency in this respect is the role of INA in the discovery of NPR1 as a central regulator of SA-dependent immune responses.

While additional screens for synthetic elicitors that are more potent and possibly distinct from those that are known are desirable, a rich arsenal of interesting plant defense-inducing compounds is already at hand. What is missing at this point, is a comprehensive systematic comparison of their functional characteristics in a single plant system, such as *Arabidopsis*. We anticipate specific interactions of many of these compounds with the plant immune system to define distinct “points of reference,” that can be probed and further examined with each compound. A next critical step will be the identification of direct synthetic elicitor targets and their roles in plant defense. This may lead to the discovery of so far unknown components of the plant immune system and reveal novel regulatory interactions controlling plant defense reactions. Furthermore, innovative screening designs are needed to complement the set of available compounds. A greater diversity of synthetic elicitors will not only be beneficial for basic research, but may also be necessary for the design of innovative multifunctional crop protectants that stimulate multiple aspects of the plant defense system and can provide resistance against a broader spectrum of plant pathogens.

## Conflict of Interest Statement

The authors declare that the research was conducted in the absence of any commercial or financial relationships that could be construed as a potential conflict of interest.
